# Therapeutic advantage of teriparatide in very elderly patients with proximal femoral fractures: a functional and BMD analysis

**DOI:** 10.1186/s12891-024-07373-6

**Published:** 2024-04-13

**Authors:** Ooi Chin Sheng, Wen-Tien Wu, Cheng-Huan Peng, Ting-Kuo Yao, Ing-Ho Chen, Jen-Hung Wang, Kuang-Ting Yeh

**Affiliations:** 1 School of Medicine, Tzu Chi University, Hualien, 970374 Taiwan; 2 Department of Orthopedics, Hualien Tzu Chi Hospital, Buddhist Tzu Chi Medical Foundation, Hualien, 970473 Taiwan; 3 Institute of Medical Sciences, Tzu Chi University, Hualien, 970374 Taiwan; 4 Department of Medical Research, Hualien Tzu Chi Hospital, Buddhist Tzu Chi Medical Foundation, Hualien, 970473 Taiwan; 5 Graduate Institute of Clinical Pharmacy, Tzu Chi University, Hualien, 970374 Taiwan

**Keywords:** Teriparatide, Bone mineral density, Barthel index, VAS score, Proximal femoral fracture

## Abstract

**Background:**

Teriparatide, a recombinant parathyroid hormone, is pivotal in osteoporosis treatment, particularly in post-surgical recovery for hip fractures. This study investigates its efficacy in functional recovery post-hip fracture surgery in elderly patients, a demographic particularly susceptible to osteoporotic fractures.

**Methods:**

In this retrospective cohort study, 150 elderly patients with proximal femoral fractures undergoing open reduction and internal fixation were enrolled. They were categorized into two groups: receiving 20 µg of daily teriparatide injections for 18 months and receiving standard antiresorptive medications during a 24-month follow-up. Detailed records of patient demographics, Fracture Risk Assessment Tool scores, and comorbidities were kept. Key outcomes, including bone mineral density (BMD) and functional scores (Barthel Index and Visual Analog Scale for hip pain), were evaluated at 3 and 24 months post-surgery.

**Results:**

Out of the original cohort, 126 patients (20 men and 106 women with an average age of 85.5 ± 9.3 years) completed the study. The teriparatide group exhibited significant enhancements in both functional scores and BMD when compared to the control group. Notably, functional improvements were less pronounced in male patients compared to female patients. Additionally, the incidence of new fractures was markedly lower in the teriparatide group.

**Conclusion:**

Administering teriparatide daily for 18 months post-surgery for proximal femoral fractures significantly benefits very elderly patients by improving functionality and bone density, with observed differences in recovery between genders. These results reinforce the efficacy of teriparatide as a potent option for treating osteoporosis-related fractures in the elderly and highlight the importance of considering gender-specific treatment and rehabilitation strategies.

## Background

Osteoporotic fractures, notably in the elderly and those with chronic conditions such as diabetes and hypertension, underscore the necessity of tailored prevention strategies, including for high-risk groups like aged individuals and postmenopausal breast cancer patients on aromatase inhibitors [1, 2]. Fragility fractures, including hip and vertebral compression fractures, pose significant risks, impairing mobility, independence, and overall quality of life, thus presenting a major public health challenge due to the associated morbidity, mortality, and healthcare costs [3–5]. Teriparatide, a parathyroid hormone analog, is recognized for its ability to expedite recovery in older adults with osteoporosis-related fractures by improving bone mineral density (BMD) and reducing pain, which could enhance activities of daily living (ADLs) [6–8]. Sun et al. found that parathyroid hormone treatment may alleviate pain and slow joint deterioration in osteoarthritis by reducing sensory nerve and vessel density through a mouse model [9]. However, adherence to teriparatide treatment can be hampered by its side effects, such as leg cramps and hypercalcemia, and concerns over injection-related discomfort, highlighting the importance of patient education and management strategies to mitigate these barriers [10]. Proximal femoral fractures, a prevalent yet catastrophic outcome of accidental falls in older adults, significantly compromise their quality of life despite surgical interventions [11]. Teriparatide, an anabolic agent, potentially facilitates recovery, enhances bone quality and reduces the risk of subsequent fractures [12].

This study aims to evaluate teriparatide’s effectiveness in relieving fracture site pain and improving ADLs, as measured by the visual analog scale (VAS) and Barthel index (BI) [13], in patients with proximal femoral fractures due to low-energy trauma, to offer insights into enhancing life quality post-fracture.

## Methods

This retrospective cohort study received approval from the hospital’s research ethics committee (Approval No: IRB108-92-B) and fully complied with relevant guidelines and regulations. We selected 150 patients admitted to our hospital for proximal femoral fractures between January 2016 and December 2020, who underwent surgical fixation. Eligible participants met the following inclusion criteria: aged 75 years or older, independent ambulation prior to fracture, fracture due to low energy trauma, successful surgical fixation without major postoperative complications (e.g., pneumonia, pulmonary embolism, complicated urinary tract infection, cerebral vascular accident, acute myocardial infarction, deep vein thrombosis), and engagement in both in-hospital and out-of-hospital rehabilitation, with continuous follow-up and anti-osteoporotic treatment using either teriparatide or denosumab for at least 24 months post-surgery. Exclusion criteria included discontinuation of anti-osteoporotic treatment, loss to follow-up, or death within 24 months post-surgery. Patients were categorized into two groups: the teriparatide group received daily Forteo injections (teriparatide 20 µg) for 18 months, while the denosumab group received Prolia (denosumab 60 mg) every six months over 24 months. A multidisciplinary rehabilitation approach, initiated immediately post-surgery and extending for at least three months post-discharge, encompassed early mobilization, strength training, balance exercises, and functional activities aimed at restoring pre-fracture functional levels and quality of life, focusing on mobility, strength, and activities of daily living [14].

We retrospectively extracted data from electronic medical records, encompassing patient demographics (age, sex, body mass index), underlying comorbidities, BMD T-scores, and a 10-year fragile fracture probability rate using the Fracture Risk Assessment Tool (FRAX). The fracture healing status, VAS for fracture site pain, and BI scores were meticulously documented. BMD measurements at the femoral neck opposite the surgical site and the average BMD at the L1–L4 lumbar spine were obtained using a dual-energy X-ray absorptiometry machine (Explorer, Hologic, Waltham, MA, USA), with readings taken at 3 and 24 months post-surgery. Similarly, VAS and BI scores were recorded at these intervals.

Treatment outcomes, including VAS and BI scores, along with BMD values for the femur and spine, were compared between patients treated with teriparatide and those receiving denosumab. The study also monitored for the occurrence of new fractures and any adverse effects from the treatments during the observation period. Data analysis was conducted using SPSS version 23.0 (IBM, Armonk, NY, USA). Baseline characteristics and comorbidities were presented as frequencies, proportions, or means ± standard deviations. We employed between-group and within-group study designs to analyze differences in four main variables (VAS and BI scores and BMD of the femur and L1–L4 spine). Between-group differences illustrated variations across distinct groups, while within-group differences highlighted variations within the same group over time. Multivariate Cox proportional hazards analysis calculated the adjusted hazard ratios (HRs) and 95% confidence intervals (CIs) for the improvement of VAS and BI scores from 3 to 24 months post-surgery, with statistical significance set at *p* < 0.05.

## Results

**Fig. 1:**
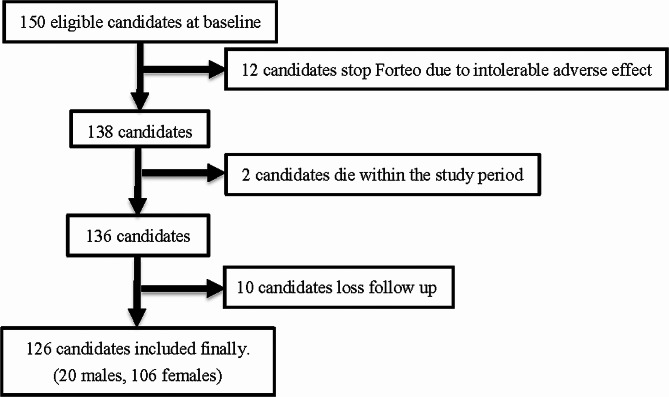
The flow chart diagram of our included patients. We initially recruited 150 patients. However, 12 of them ceased teriparatide treatment because of intolerable adverse effects, another 2 of them died as a result of cardiac problems, and another 73 of them were lost of follow-up. Finally, we included a total of 63 patients for further study

We initially recruited 150 patients (30 men and 120 women). However, 12 candidates ceased Forteo treatment because of intolerable adverse effects, including general edematous change, upper limb pain and swelling, residual numbness, bilateral knee pain with numbness and swelling, constipation, decreased appetite, headache, nausea, and allergic skin reactions. During the study period, another two patients died from cardiac problems. Although teriparatide has been suggested to influence heart rhythm potentially, our patient’s death was primarily attributed to a cardiac issue, specifically myocardial infarction resulting from atherosclerosis. Of the remaining 136 patients, 10 were lost to follow-up. Ultimately, our study analyzed 126 patients (20 men and 106 women) with a follow-up period of 24 months (Fig. 1).

68 of the patients received Forteo, and the other 58 of them received Prolia for postoperative anti-osteoporotic medication. The average age of participants was 85.5 ± 9.3 years, with no significant difference between groups (*p* = 0.730). The distribution of male (15.9%) and female (84.1%) patients across groups also showed no significant difference (*p* = 0.319). BMI averaged 22.3 ± 3.8 across the cohort, indicating no significant difference between the Forteo and Prolia groups (*p* = 0.792) (Table 1). FRAX scores for major and hip fractures, 3-month postoperative VAS for pain, Barthel scale scores for functionality, and BMD measurements at the femur and L1-L4 spine, were comparable between groups, showing no significant differences. Notably, the occurrence of new fractures was significantly lower in the Forteo group (2.9%) compared to the Prolia group (10.3%), with a *p*-value of 0.042. Another significant finding was the fracture healing period, which was shorter for the Forteo group (12.4 ± 3.8 months) compared to the Prolia group (18.2 ± 5.1 months), with a *p*-value of 0.023, the demographic analysis revealed an average age of 85.5 ± 9.3 years with a majority female population (84.1%) and no significant differences in age, sex distribution, BMI, or FRAX scores between patients treated with Forteo and those in the Prolia group. Notably, the occurrence of new fractures during the study period was significantly lower in the Forteo group at 2.9% compared to 10.3% in the Prolia group (Table 1).

**Table 1:** Demographics of patients receiving hip fracture fixation surgery (*n* = 126)

Variables	Forteo	Prolia		Total	*p*-value
N	68	58		126	
Age	85.8 ± 9.2	85 ± 9.5		85.5 ± 9.3	0.730
Sex	-	-		-	0.319
Male	14 (2.6%)	6 (1.3%)		20 (15.9%)	
Female	54 (79.4%)	52 (89.7%)		106 (84.1%)	
BMI	22.4 ± 4.3	22.2 ± 3.3		22.3 ± 3.8	0.792
FRAX major	26.7 ± 9.9	27.9 ± 11.2		27.3 ± 1.5	0.664
FRAX hip	14.8 ± 7.7	16.0 ± 1.6		15.4 ± 9.1	0.606
VAS(PostOP 3 months)	7.3 ± 0.9	7.2 ± 0.9		7.3 ± 0.9	0.717
Barthel scale (PostOP 3 months)	33.1 ± 9.9	38.8 ± 1.2		35.7 ± 1.3	0.067
BMD Femur (PostOP 3 months)	-3.0 ± 0.6	-3.1 ± 0.9		-3.1 ± 0.7	0.749
BMD L1-4 (PostOP 3 months)	-3.1 ± 0.9	-3.2 ± 1.4		-3.1 ± 1.1	0.747
Previous medication	-	-		-	0.061
None	4(5.9%)	16 (27.6%)		20 (15.9%)	
Prolia	34 (5.0%)	24 (41.4%)		58 (46.0%)	
Bisphosphonate	30 (44.1%)	18 (31.0%)		48 (38.1%)	
Comorbidity	-	-		-	
Dyslipidemia	6 (8.8%)	10 (17.2%)		16 (12.7%)	0.453
HTN (%)	40 (58.8%)	44 (75.9%)		84 (66.7%)	0.153
DM (%)	6 (8.8%)	16 (27.6%)		22 (17.5%)	0.051
CKD (%)	10 (14.7%)	8 (13.8%)		18 (14.3%)	1.000
CAD (%)	10 (14.7%)	4 (6.9%)		14 (11.1%)	0.437
Outcome	-	-		-	
New fracture occurrence (%)	2 (2.9%)	6 (10.3%)		8 (6.3%)	0.042*
Tolerable side effects (%)	6 (8.8%)	3 (5.2%)		9 (7.1%)	0.062
Fracture healing period (M)	12.4 ± 3.8	18.2 ± 5.1		15.1 ± 4.3	0.023*

Data are presented as n or mean ± standard deviation. *p-value < 0.05 was considered statistically significant after test.

*FRAX* Fracture Risk Assessment Tool, *VAS* visual analogue scale, *BMD* bone mineral density, *DM* diabetes mellitus, *CKD* chronic kidney disease, *CAD* coronary artery disease. M: months

Functional improvements were significant in patients receiving Forteo, with VAS scores showing a marked decrease from 7.3 ± 0.9 to 2.8 ± 0.7 and BI scores improving from 33.1 ± 9.9 to 80.3 ± 12.6 over the 24-month postoperative period. This contrasted with the Prolia group, where VAS scores decreased from 7.2 ± 0.9 to 4.2 ± 0.9 and BI scores from 38.8 ± 1.2 to 69.0 ± 1.1, indicating a more substantial functional recovery in the Forteo group. BMD measurements further supported the therapeutic benefits of Forteo, with improvements observed in femur BMD from − 3.0 ± 0.6 to -2.3 ± 0.6 and L1-L4 spine BMD from − 3.1 ± 0.9 to -2.1 ± 1.3, compared to less pronounced improvements in the Prolia group (Table 2).

**Table 2:** Function score improvement of patients receiving different anti-osteoporotic medications after surgery for fracture (*n* = 126)

Item	Medication	*N*	Postop 3 months	Postop 24 months		Diff		Between-Group *p*-value	Within-Group *p*-value
VAS	Forteo	68	7.3 ± 0.9	2.8 ± 0.7		-4.4 ± 1.2		0.020*	< 0.001*
	Prolia	58	7.2 ± 0.9	4.2 ± 0.9		-3.1 ± 1.1			< 0.001*
Barthel scale	Forteo	68	33.1 ± 9.9	80.3 ± 12.6		46.2 ± 7.5		< 0.001*	< 0.001*
	Prolia	58	38.8 ± 1.2	69.0 ± 1.1		31.2 ± 6.6			< 0.001*
BMD Femur	Forteo	68	-3.0 ± 0.6	-2.3 ± 0.6		0.7 ± 0.5		0.002*	< 0.001*
	Prolia	58	-3.1 ± 0.9	-2.6 ± 0.7		0.5 ± 0.7			< 0.001*
BMD L1-4	Forteo	68	-3.1 ± 0.9	-2.1 ± 1.3		0.9 ± 0.8		0.003*	< 0.001*
	Prolia	58	-3.2 ± 1.4	-2.6 ± 1.3		0.6 ± 0.7			< 0.001*

Data are presented as n or mean ± standard deviation. *p-value < 0.05 was considered statistically significant after test. *VAS* visual analogue scale, *BMD* bone mineral density

**Table 3:** Analysis of the factors associated with improvement of functional score among the patients receiving proximal femoral fracture fixation surgery (*n* = 126)

Item	**Diff. of VAS (PostOP3M-PostOP24M)**				**Diff. of BI (PostOP24M-PostOP3M)**						
	**Crude**		**Adjusted**		**Crude**		**Adjusted**				
	**β (95% CI)**	***p*** **-value**	**β (95% CI)**	***p*** **-value**	**β (95% CI)**	***p*** **-value**		**β (95% CI)**	***p*** **-value**		
Age	0.00 (-0.03, 0.03)	0.868	0.01 (-0.02, 0.04)	0.614	-0.10 (-0.30, 0.09)	0.284		-0.07 (-0.26, 0.12)	0.447		
Sex (Male vs. Female)	-1.04 (-1.81, -0.27)	0.009*	-1.11 (-2.01, -0.20)	0.018*	-2.14 (-2.32, -0.74)	0.021*		-1.66 (-2.14, -0.60)	0.016*		
BMI	0.00 (-0.08, 0.08)	0.930	-0.05 (-0.13, 0.04)	0.272	-0.07 (-0.55, 0.40)	0.764		0.38 (-0.18, 0.93)	0.181		
Medication (Forteo vs. Prolia)	4.76 (-0.30, 9.82)	0.002*	2.07 (1.50, 4.65)	0.002*	3.91 (0.90, 8.71)	0.009*		6.80 (1.04, 12.56)	0.021*		
FRAX major	0.01 (-0.01, 0.04)	0.331			0.07 (-0.10, 0.24)	0.407					
FRAX hip	0.01 (-0.02, 0.04)	0.600			0.10 (-0.09, 0.30)	0.302					
Previous medicine	-	-			-	-		-	-		
None	Ref.	Ref.			Ref.	Ref.		Ref.	Ref.		
Prolia	0.61 (-0.23, 1.45)	0.154			-0.11 (1.70, 2.49)	0.720		0.95 (-0.37, 1.27)	0.067		
Bisphosphonate	-0.02 (-0.88, 0.85)	0.969			1.38 (-3.82, 6.57)	0.598		-0.15 (-5.65, 5.35)	0.956		
Dyslipidemia vs. None	-0.57 (-1.45, 0.31)	0.200			-1.53 (-6.91, 3.84)	0.570					
HTN vs. None	0.31 (-0.32, 0.94)	0.327			0.36 (-3.45, 4.16)	0.852					
DM vs. None	0.26 (-0.52, 1.04)	0.501			-2.52 (-7.20, 2.16)	0.286					
CKD vs. None	-0.63 (-1.46, 0.20)	0.136			3.06 (-2.01, 8.12)	0.232					
CAD vs. None	-0.98 (-1.89, -0.07)	0.035*	-0.79 (-1.80, 0.22)	0.123	-2.41 (-8.09, 3.26)	0.399					

Data are presented as Odds ratio (95% CI). *p-value < 0.05 was considered statistically significant after test.

*BI* Barthel index, *FRAX* Fracture Risk Assessment Tool, *DM* diabetes mellitus, *CKD* chronic kidney disease, *CAD* coronary artery disease

Further analysis highlighted those males showed significantly greater improvement in both VAS and BI scores compared to females, with adjusted β indicating a more considerable improvement for males in VAS (-1.11) and BI (-1.66), both with *p* < 0.05, while teriparatide was significantly associated with greater improvements in VAS and BI scores compared to Prolia, with adjusted β showing notable improvements in both VAS (2.07) and BI (6.80), *p* < 0.05. Age, BMI, and other demographic factors were not significantly associated with these functional improvements (Table 3).

## Discussion

Our study results revealed the effectiveness of teriparatide in promoting recovery and improving bone quality after hip fracture surgery. While baseline demographics and clinical measures were similar between the two groups, patients treated with teriparatide had a lower incidence of new fractures and a shorter healing period compared to those treated with Prolia, indicating potential differences in postoperative recovery and efficacy between the two medications. The results are consistent with the evidence presented in recent literature, which underscores the differential impact of anti-osteoporotic medications on fracture risk reduction and healing processes. For instance, a study by authors in Therapeutic Advances in Musculoskeletal Disease [15] emphasizes the role of teriparatide in enhancing bone formation and accelerating fracture healing, which aligns with our observation of a reduced incidence of new fractures and shorter healing periods in the Forteo group. Similarly, research published in the Journal of Bone and Mineral Research [16] discusses the anabolic effects of teriparatide on bone, supporting our findings that Forteo may facilitate quicker recovery from hip fractures. Furthermore, an article in the Journal of Functional Morphology and Kinesiology [17] reviews the mechanisms by which teriparatide and denosumab differentially affect bone health, with teriparatide showing superior outcomes in bone regeneration and fracture healing. Our study’s results, indicating a lower new fracture occurrence and a shorter fracture healing period in patients receiving Forteo, reflect the broader evidence suggesting that the anabolic action of teriparatide not only improves bone density but also enhances the structural integrity and healing capacity of bone more effectively than the antiresorptive mechanism of denosumab.

Changes in scores and BMD became significant after 18 doses of teriparatide treatment; thus, teriparatide appears effective for healing proximal femoral fractures. Our results supported our hypothesis that teriparatide intervention leads to less pain and more favorable functional outcomes. Other studies have discussed the benefits of teriparatide. Dempster et al. found that the cancellous mineralizing surface–bone surface ratio was significantly higher in the Forteo group than in the Prolia group after three months [18]. Furthermore, bone formation decreased after denosumab treatment but increased after teriparatide treatment. The European Forsteo Observational Study of Graeff et al. reported that teriparatide treatment increased initial stiffness among patients by 25% after approximately 24 months [19]. Moreover, the maximum moment and maximum force of patients’ vertebral bodies exhibited nearly identical changes, and the osteoblastic effect was sustained and became more significant after a longer teriparatide intervention. Additionally, a fracture prevention trial revealed a significant reduction in vertebral fracture risk after teriparatide treatment was discontinued for at least 18 months among patients who had received teriparatide for 19 months on average [20]. This finding supports our results, which indicated that teriparatide reduces pain and improves functional outcomes one month after the cessation of treatment. A randomized control trial conducted by Malouf-Sierra et al. revealed that patients who received teriparatide for 78 weeks had significantly higher BMD in the lumbar spine and contralateral unfractured femoral neck compared with those who received risedronate [21]. This finding was further supported by the 2018 VERO study, which reported that postmenopausal women with osteoporosis who received teriparatide treatment had a lower risk of new vertebral and clinical fractures compared with those who received risedronate [22]. In addition, according to the findings presented in the ALAFOS study from Chen et al. in 2021 postmenopausal women diagnosed with osteoporosis, deemed at an increased risk of fractures, and administered teriparatide as part of standard clinical practice, exhibited a significant reduction in fragility fracture rates within the initial 6 months of treatment. Furthermore, these women reported enhancements in back pain and Health-Related Quality of Life [23]. In a study conducted by Kim et al. in 2019 revealed that teriparatide therapy demonstrated a significant increase in Harris Hip Score and a decrease in Visual Analog Scale pain scores. The mean time to fracture healing post-operatively was notably reduced in the teriparatide-treated groups. Furthermore, the frequency of patients reporting postoperative complications showed a marked decrease in the teriparatide-treated groups. The study suggests that short-term daily teriparatide use for osteoporosis treatment improves radiographic fracture healing in hip fractures and reduces complication rates [24].

In our study, male patients demonstrated a lower degree of functional improvement following fixation surgery for proximal femoral fractures than their female counterparts, a finding that aligns with existing literature. This gender disparity could be attributed to various factors. Biologically, men typically have higher peak bone mass; however, when osteoporosis is present, it often indicates more severe pathology, potentially impacting recovery [25]. Additionally, hormonal differences, particularly the lack of estrogen’s protective effect in men, may influence bone healing and functional recovery [26]. Socio-behavioral aspects also contribute to this disparity. Men generally exhibit less health-seeking behavior and lower adherence to postoperative care [27], and lifestyle factors like smoking or lower physical activity levels prevalent in men can adversely affect recovery outcomes [28]. These findings underscore the need for tailored postoperative management strategies that address the unique biological and socio-behavioral needs of male patients to optimize recovery outcomes.

Minor side effects experienced by patients during the injection treatment, such as discomfort and changes in feelings, which may affect their ability to continue treatment, are often overlooked by family members and physicians; however, such side effects may influence improvements in functional outcomes. The European Forsteo Observational Study reported that the fracture risk subsided during teriparatide treatment but provided no evidence of changes in fracture risk after teriparatide was discontinued [29]. Improvements in back pain continued for more than 18 months after teriparatide discontinuation. Our study found only lower VAS and higher BI scores one month after teriparatide discontinuation. However, these benefits may last for more than 18 months. Silverman et al. also reported significant decreases in the hip fracture rate after more than 18 months of teriparatide treatment [30]. Nevertheless, further research is necessary to verify this finding.

This study had some limitations. First, the sample size was relatively small, especially the number of male patients. Thus, further studies are necessary to compare the effects of teriparatide intervention between male and female patients. Second, there was no information regarding the factors such as patient’s care quality, rehabilitation condition after postoperative three months, or dietary habits at home from the medical charts, which may affect patients’ functional status and bone density. Third, no significant differences in demographic data were observed between the two groups; however, fear of injections or the inconvenience of the treatment from the patient’s perspective may have led to selection bias (e.g., patients choosing different medications). Lastly, another limitation of this retrospective study was the risk of selection bias, given that participant inclusion was based on historical records rather than a randomized sampling approach. This methodological choice could potentially curtail the applicability of our results to a broader population. In despite of these limitations, our study also had several notable strengths. First, we included FRAX scores and several comorbidities in the demographic data to ensure that the influencing factors could be minimized. Moreover, we recorded VAS and BI scores to examine functional outcomes in older adult patients. Our results revealed that an 18-month course of teriparatide injection treatment may be more effective in improving the quality of life and bone quality of patients aged more than 75 years with proximal femoral fracture and who underwent fixation surgery compared with other medications. Such improvements are crucial for addressing the health of older adults in our aging society. Further studies are recommended to focus on decreasing the discomfort and inconvenience of teriparatide injections and comparing teriparatide with other medications.

## Conclusion

In conclusion, our study underscores the efficacy of teriparatide in promoting faster recovery and reducing the likelihood of subsequent fractures among elderly patients following hip fracture fixation surgery, compared to denosumab. The observed differences in fracture healing periods and new fracture occurrences highlight the importance of selecting appropriate anti-osteoporotic therapy to optimize post-surgical outcomes. These insights contribute to the growing body of evidence favoring teriparatide in the postoperative management of hip fractures, reinforcing the need for healthcare providers to consider individual patient profiles and fracture risks when devising treatment plans. Future research should continue to explore the comparative effectiveness of various anti-osteoporotic agents further to refine post-surgical care strategies for geriatric patients with proximal femoral fractures.

## Data Availability

The datasets used and/or analyzed during the current study are available from the corresponding author on reasonable request.

## Abbreviations

ADLs: Activities of daily living
BI: Barthel index
BMD: Bone mineral density
CIs: Confidence intervals
FRAX: Fracture Risk Assessment Tool
HRs: Hazard ratios
VAS: Visual analog scale
